# Impact of Bark-Sourced Building Blocks as Substitutes for Fossil-Derived Polyols on the Structural, Thermal, and Mechanical Properties of Polyurethane Networks

**DOI:** 10.3390/polym15173503

**Published:** 2023-08-22

**Authors:** Alexandr Arshanitsa, Jevgenija Ponomarenko, Matiss Pals, Lilija Jashina, Maris Lauberts

**Affiliations:** Latvian State Institute of Wood Chemistry, Dzerbenes Street 27, LV-1006 Riga, Latvia; jevgenijaponomarenko@inbox.lv (J.P.); matiss.pals@kki.lv (M.P.); lilija_jasina@inbox.lv (L.J.); marislauberts@gmail.com (M.L.)

**Keywords:** bark, macromonomer, phenolic groups, aliphatic OH, polyurethane network, tensile properties, thermal oxidative stability

## Abstract

The hydrophilic extractives isolated from black alder (*Alnus glutinosa*) bark through hot water extraction were characterized as novel renewable macromonomers capable of forming polyurethane (PU) networks based on a commercial polyisocyanate, with partial or complete replacement of petroleum-derived polyol polyether. The bark-sourced bio-polyol mainly consists of the xyloside form of the diarylheptanoid oregonin, along with oligomeric flavonoids and carbohydrates, resulting in a total OH group content of 15.1 mmol·g^−1^ and a molecular weight (M_n_) of approximately 750 g∙mol^−1^. The ^31^P NMR data confirmed a similar proportion of aliphatic OH and phenolic groups. Three-component PU compositions were prepared using polyethylene glycol (M_n_ = 400 g∙mol^−1^), bio-polyol (up to 50%), and polymeric diphenylmethane diisocyanate, which were pre-polymerized in tetrahydrofuran (THF) solution with tin organic and tertiary amine catalysts. The resulting mixture was cast and subjected to thermal post-curing. Calculation and experimental data confirmed the crosslinking activity of the bark-sourced bio-polyol in PU, leading to an increase in glass transition temperature (Tg), a decrease in sol fraction yield upon leaching of cured PU networks in THF, a significant increase in Young’s modulus and tensile strength. The macromonomers derived from bark promoted char formation under high temperature and oxidative stress conditions, limiting heat release during macromolecular network degradation compared to bio-polyol-free PU. It was observed that amine catalysts, which are active in urethane formation with phenolic groups, promoted the formation of PU with higher Tg and modulus at tensile but with less limitation of heat liberation during PU macromolecular structure degradation. The high functionality of the bark-derived bio-polyol, along with the equal proportion of phenolic and aliphatic OH groups, allows for further optimization of PU characteristics using three variables: increasing the substitution extent of commercial polyethers, decreasing the NCO/OH ratio, and selecting the type of catalyst used.

## 1. Introduction

Polyurethane (PU) is one of the most versatile classes of polymeric materials, with its exploitation characteristics capable of being controlled within a wide range by varying the structure and proportion of its main components, including isocyanate, polyols, and catalysts, as well as the processing regimes used [1]. PU products encompass foams, elastomers, adhesive constructive materials, coatings, and more, making them applicable in practically all branches of modern industry [2]. In 2021, the global market volume of PU amounted to approximately 24.7 million tons, and it is projected to grow to 29.2 million tons by 2029 [3].

Similar to widely used plastics such as polyethylene, polyvinyl chloride, and polystyrene, the raw materials for PU are derived from petroleum resources. However, the non-biodegradability of fossil-based PU and the depletion of petroleum resources have raised serious economic and environmental concerns. As a result, the PU industry is driven to increase the renewable content in PU [2,4].

An approach aimed at obtaining isocyanate-free polyurethane (PU) has recently been developed, which involves the carbonation of tannin or lignin with dimethyl carbonate followed by condensation with diamines. This method enables the production of PU with a renewable content of up to 85% [5]. However, it is important to note that this approach has only been developed at the laboratory scale, and a comparison of the properties between hydroxyl-containing PU obtained through alternative methods and traditional isocyanate-based PU has not yet been studied. Therefore, it is recognized that the most attractive approach for today’s industry to produce bio-based PU is the partial or complete substitution of fossil-based polyols with renewable alternatives while retaining fossil-originated polyisocyanate in the PU composition [6].

Lignocellulosic biomass, including wood, bark, cereal crops, and their constituents such as lignin, carbohydrates, and extractives [7], has been introduced into polyurethane (PU) as hydroxyl-containing renewable compounds, aiming to achieve a more environmentally friendly PU with functional characteristics similar to conventional PU. Lignin, as the first studied lignocellulosic component, has been carefully examined for its application in PU composites as a macromonomer [8]. Detailed investigations on the effects of different non-chemically modified lignins, including kraft, organosolv, and lignosulphonates, on the properties of PU elastomers and foams have been performed [8,9,10,11,12,13,14,15]. It has been shown that lignin, when dissolved in aprotic solvents along with commercial polyol and isocyanate, can be introduced into the PU network as a macromonomer, resulting in increased crosslink density, glass transition temperature, and mechanical characteristics of the PU networks. Chemical modification of lignin through oxypropylation allows the production of lignin-based polyols with comparable characteristics to commercial polyol polyethers [16].

However, oxypropylation was accompanied by the homopolymerization of propylene oxide, resulting in a decrease in the biomass content in the polyol below 40%. Bark, which is a multi-tonnage waste product of forestry with annual production between 300–400 million m^3^ [17], is another highly available lignocellulosic raw material that has been tested as a renewable polyol to replace commercial polyol in PU foam compositions [18,19,20,21]. In the process, the initial bark or previously isolated tannins were transformed into liquid polyol through liquefaction using polyhydric alcohols such as propylene glycols or polyethylene glycols [21] or through oxyalkylation [18]. Both processes involved the use of fossil-derived polyhydric alcohols and propylene oxide, resulting in a renewable content in the obtained polyol in the range of 13.5–38% [22]. This led to a decrease of more than twofold in the content of biomass in the PU foams. The low content of biomass in the final material and the high temperature of biomass modification, especially in the process of oxypropylation, are the main disadvantages of the aforementioned processes of lignin and bark modification.

Furthermore, tree bark is known to be enriched with hydrophilic low molecular compounds of predominantly phenolic origin with admixtures of carbohydrates enriched with OH groups. These compounds can be easily isolated from biomass using green extraction methods and could be used as a natural bio-polyol without the need for deep modification [23].

During this work, the novel bark-sourced bio-polyol was isolated through short-time microwave-assisted water extraction of black alder bark at 90 °C [24,25,26]. This bio-polyol mainly consists of xyloside derivatives of diarylheptanoids, which possess both aliphatic hydroxyl (OH) and phenolic groups [26]. Unlike tannins, which are the most widely used bark extractives and have an aromatic and aliphatic cyclic structure, diarylheptanoids have C7 aliphatic linear chains that can act as natural soft segments in the PU network. This could provide a favorable balance between rigidity and flexibility in PU. The study aimed to evaluate the effect of full and partial replacement of fossil-sourced polyol with the bark-sourced alternative, considering the bio-polyol content in the PU formulation of up to 50%, on the structural, thermal, and mechanical characteristics of the PU networks. Additionally, the utilization of the bark-sourced polyol as a crosslinking agent and promoter of charcoal formation under thermal oxidative conditions in the PU network was considered. For the direct introduction of the bark-sourced polyol into the PU network without modification, two catalysts, tin organics and tertiary amines, which are widely used in PU processing and exhibit different selectivity towards the interaction of aliphatic OH and phenolic groups with isocyanates, were tested.

## 2. Materials and Methods

### 2.1. Materials

The tetrahydrofuran (THF)-soluble fraction of extractives previously isolated from black alder (*Alnus glutinosa*) bark by microwave-assisted water extraction was used as a natural polyol to gradually substitute fossil-derived polyethylene glycol PEG 400 in PU elastomers. Commercial polymeric diphenylmethane diisocyanate (PMDI) with [NCO] = 7.5 mmol·g^−1^ and an average functionality of 2.7 was purchased from BASF (Ludwigshafen, Germany). 1,4-diazabicyclo[2.2.2]octane (DABCO), dibutyltin dilaurate (DBTDL) with a purity of ≥95%, anhydrous THF with a purity of ≥99.9%, PEG 400 with a purity of ≥95%, acetonitrile (hyper grade for LC-MS LiChrosolv^®^), oregonin with a purity of ≥95% (LC/MS-ELSD), and hirsutenone with a purity of ≥95% (LC/MS-ELSD) were purchased from Merck (Darmstadt, Germany) and used without further purification.

### 2.2. Preparation of Bark-Sourced Polyol

The process of preparing the bio-polyol involved isolating black alder bark extractives using hot water (90 °C) in a specifically engineered MW extractor [25]. The yield of extractives obtained was about 15% based on the dry weight of black alder bark. Following the extraction, fractionation was performed to obtain a bio-polyol suitable for producing PU elastomers using the casting method, with tetrahydrofuran (THF) as the solvent. The THF-soluble fraction of the extractives, with a yield of 62 ± 1%, was isolated and studied as the bio-polyol. To fractionate the extract, the lyophilized extract was dissolved in THF at a solid-to-liquid ratio of 1/10 (*w*/*v*), and the remaining solid was separated using a glass filter. The solvent was then distilled off, and the resulting mixture was dissolved in water and subsequently lyophilized.

### 2.3. Composition of Bark-Sourced Polyol

#### 2.3.1. UPLC-ELSD Chromatography

The composition of the bio-polyol was analyzed using UPLC-ELSD chromatography. For this analysis, an Acquity UPLC system manufactured by Waters Corp., based in Singapore, was used. The system was equipped with photodiode array (PDA) and evaporative light scattering (ELS) detectors, also from Waters Corp. In this study, a Waters Acquity UPLC column (2.1 × 100 mm i.d., 1.7 μm, CSH C18) was employed for the separation of components. The eluent used in the system consisted of water containing 0.1% formic acid (A) and acetonitrile (B). During the analysis, a gradient solvent system, as described in Table 1, was utilized. The separation process was carried out at a flow rate of 0.5 mL∙min^−1^. Used solvent gradient system (solvent A and solvent B) was developed based on our experience and preliminary investigation of these samples. Used solvent gradient system was controlled using Empower software 3.8.0.

#### 2.3.2. Total Polyphenolic Content

The total polyphenolic content (TPC) was determined and expressed as grams of gallic acid equivalent (GAE) per unit weight of produced bio-polyol. The analysis was conducted using the Folin–Ciocalteu method as described by [27]. The measurement involved detecting the intensity of the blue complex formed by the interaction of an ethanol solution of extractives with the Folin–Ciocalteu reagent. A PerkinElmer Lambda 650 UV/VIS spectrophotometer (Perkin Elmer, Waltham, MA, USA) was used to measure the absorbance at 765 nm against the blank, with gallic acid serving as the standard. The averaged data of three repeated experiments were presented with standard deviation.

#### 2.3.3. Proanthocyanidins Content

The total content of proanthocyanidins (PAC) in the bio-polyol was determined using the acid-butanol method, as described by Hagerman [28]. The averaged data of three repeated experiments were presented with standard deviation.

#### 2.3.4. Monomeric Sugar Content

The total monomeric carbohydrate content in the bark-derived polyol, both without and with prior H_2_SO_4_ hydrolysis, was determined using the gas chromatography with flame ionization detection (GC-FID) method, following the procedures described elsewhere [29,30]. The analysis was conducted using an Agilent 6850 series GC instrument (Agilent, Santa Clara, CA, USA) equipped with a 30 m DB-1701 column. The averaged data of three repeated experiments were presented with standard deviations.

### 2.4. Functionality of Bark-Sourced Polyol

#### 2.4.1. Wet Chemistry Analysis

The content of various OH groups in the bio-polyol under study was determined following the procedures described in the monograph by Zakis [31]. The total content of aliphatic and phenolic groups in the biomass was measured through acetylation of the extractives at 40 °C for 24 h, followed by potentiometric titration of the excess acetic acid using 0.1 M NaOH. The cumulative content of acidic groups, including phenolic and carboxylic groups, was determined by back conductometric titration of the alkali solution of the extractives with 0.1 M HCl. This was carried out using an automatic titration device ABU 910, coupled with a Conductometer CDM 210 and Titration Manager TIM900 (Radiometer, Copenhagen, Denmark). To determine the carboxyl groups separately, the calcium-acetate chemisorption method was employed.

The aliphatic OH content in the bio-polyol was calculated based on the experimental data obtained as follows: OH_aliphatic_ = OH_acetylated_ + OH_COOH_ − OH_phenolic_ [32]. Each experiment was conducted in triplicate, and the mean and standard deviation were calculated.

#### 2.4.2. ^31^P NMR

Bark-derived polyol was analyzed in duplicate using ^31^P NMR according to [33]. The 2-chloro-4,4,5,5-tetramethyl-1,3-2-dioxaphospholan (TMDP) and of N-hydroxy-5-norbornene-2,3-dicarboximide and 5 mg·mL^−1^ were used as phosphorylation agent and internal standard correspondingly. The NMR spectra were recorded on a 600 MHz Bruker Biospin (Rheinstetten, Germany, BASIC PROBHD) with the following program: spectral width—100 p.p.m., acquisition time—0.8 s, relaxation time ≥ 10 s, and scan number 128.

#### 2.4.3. Gel Permeation Chromatography

The molecular mass distribution (MMD) of the bark-sourced polyol was analyzed using an Agilent 1260 Infinity HPLC system (Agilent, Santa Clara, CA, USA) equipped with a Plgel Mixed-E 300 × 8 mm column and a refractive index (RI) detector Optilab^®^, which was thermostated at 40 °C. The samples were dissolved in THF to obtain a concentration of 20 mg·L^−1^. An injection volume of 100 µL was used. THF was employed as the mobile phase with a flow rate of 1.0 mL·min^−1^. Sodium-polystyrene sulfonates were used as the calibration standard for MMD, with a specified molecular weight range of 500–20,000 Da [34].

### 2.5. Synthesis of PU Networks

PU networks in the form of films with a thickness of 0.200–0.250 mm were prepared by casting three-component PU systems. These systems consisted of bark-derived bio-polyol, PEG 400, and pre-polymerized PMDI, which were dissolved in extra dry THF. The bio-polyol content in the PU varied from 3% to 30% with an NCO/OH ratio of 1.0. For one particular sample, the NCO/OH ratio was reduced to 0.5 in order to achieve a 50% content of the bio-based component in the PU. DBTL or DABCO catalysts were used at equivalent concentrations of 2% (*w*/*w*) and 0.18% (*w*/*w*), respectively, based on the total weight of isocyanate and polyols. Each PU composition was pre-polymerized for 20–30 min followed by a 2-min treatment in an ultrasound bath. Subsequently, 45 g of the solution containing approximately 10.0 g of the prepolymer was poured into a Teflon mold and covered with a flat glass plate. After several hours, the cover was removed, and slow solvent evaporation took place overnight. The resulting films were then transferred to a desiccator filled with P_2_O_5_ for further 5 days of curing at 25 °C, followed by post-curing in an oven at 90 °C for 8 h, as described in previous works [9,12].

### 2.6. Characterization of PU Networks

#### 2.6.1. FTIR Spectra

The structure of the PU networks was studied using the attenuated total reflectance (ATR)–FTIR method. Measurements were performed using a Spectrum One spectrometer (Perkin Elmer, Waltham, MA, USA) equipped with an FTIR-ATR ZnSe cell. The spectral range analyzed was 4000–500 cm^−1^. A total of 32 scans were acquired at a resolution of 4 cm^−1^. Baseline correction was conducted using the Spectrum version 5.0 software (Perkin-Elmer). The resulting spectra were normalized to the sum intensity of all peaks. Duplicate analyses were performed, and the average data were obtained for further analysis.

#### 2.6.2. Glass Transition Temperature (Tg) by Differential Scanning Calorimetry (DSC)

The Tg of the PU networks was measured using a Mettler Toledo DSC 823e device (Mettler Toledo, Greinfensee, Switzerland). To eliminate the effect of enthalpy relaxation, a sample weighing approximately 8.0 mg was heated from 25 °C to 180 °C at a rate of 10 °C per minute, followed by cooling from 180 °C to −50 °C. This heating and cooling scan was repeated twice. The Tg, defined as the inflection point, and the corresponding heat capacity gap (ΔCp) were determined from the second scan using the Mettler Toledo software STARe SW V 16.10. Three repetitions were performed for each sample, and the average data were used for further discussion.

#### 2.6.3. Non-Isothermal TG/DTG/DSC of PU Network in Air

Non-isothermal thermal analysis in the air was performed on a PU sample weighing approximately 20 mg using the Seteram Setline device (Seteram, Caluire-et-Coire, France). The temperature range was set from 25 °C to 700 °C with a heating rate of 5 °C per minute. The Calisto 2.0 software was utilized to establish the baseline for heat flow and perform integration. The TG and DSC data were employed to calculate the PU conversion based on weight loss and heat released, respectively. Each analysis was repeated three times, and the mean and standard deviation were used to analyze the results.

#### 2.6.4. Tensile Tests of PU Networks

The tensile properties of PU films were determined in accordance with ASTM D882-12 standard [35] using the ZWICK/RoellZ100 universal testing machine (Zwick/Roell, Ulm, Germany) The tests were conducted at a temperature of 21 °C and a relative humidity of 60%. The dimensions of the samples were as follows: length—100 mm, width—5.0 mm, and thickness—0.200–0.250 mm. The crosshead distance was set to 70 mm. For each sample, six to eight specimens were tested. The Young modulus, strength at break, and elongation at break were calculated using the ZWICK software TestXpert II 3.3.

#### 2.6.5. The Sol Fraction Content in Cured PU Networks

The oven-dried PU films, weighing approximately 1.0 g, were placed in glass dishes containing THF. The amount of THF added depended on the weight of the films to achieve a film-solvent ratio of 1:15 (*w*:*v*). The dishes containing the immersed PU films were sealed and regularly mixed for a duration of 7 days. After this period, the PU films were carefully removed from the solvent, dried, and weighed again. Five parallel tests were conducted for each sample. The sol fraction content of the PU films was determined by calculating the average weight loss as a percentage relative to the initial sample weight.

## 3. Results

### 3.1. Composition and Functionality of Black Alder Bark-Sourced Bio-Polyol

In this study, the extractives isolated from black alder bark were considered as a mixed bio-polyol that could be incorporated into a three-component PMDI-based PU network by partially or completely substituting the commercial polyol polyether PEG 400. To perform copolymerization and obtain PU through casting methods, it was necessary for all components to be completely soluble in THF. Therefore, the raw extract was fractionated with THF, and the soluble fraction of the raw extract was used. The analysis showed that polyphenolics were the dominant component of the THF-soluble fraction, and their content in the biomass increased due to the concentration effect. The total phenolic content (TPC) in the THF-soluble fraction, raw extractives, and THF-insoluble fraction was 0.72 ± 0.10 GAE g·g^−1^, 0.54 ± 0.05 GAE g·g^−1^, and 0.21 ± 0.01 GAE g·g^−1^, respectively. Additionally, the content of proanthocyanidins (PAC), which are oligomers of flavonoids, analyzed by the acid butanol method, increased in the same range, and consisted of 22.8 ± 1.8%, 12.1 ± 1.0% and 6.5 ± 0.5%, respectively. In contrast, the carbohydrate content decreases in the following order: THF-insoluble fraction > raw extract > THF-soluble fraction (Table 2).

**Figure 1:**
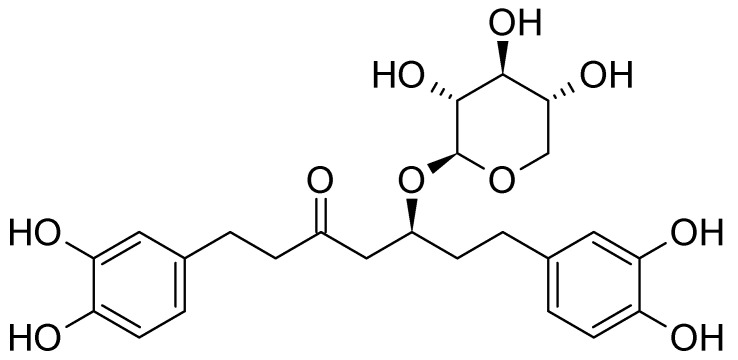
The structural formula of oregonin.

The liquid chromatography results confirm the high polyphenolic content in the THF-soluble fraction, which includes diarylheptanoids, proanthocyanidins, and a mixture of organic acids and carbohydrates (Figure S1, Table S1). Using UHPLC-ELSD with commercial oregonin as the internal standard for calibration, it was demonstrated that 74 ± 3% of the THF-soluble fraction comprises oregonin (Figure 1).

This glucoside form of the diarylheptanoid, which possesses both aliphatic and phenolic hydroxyl groups capable of forming urethane bonds through condensation with isocyanates, can be considered a natural macromonomer suitable for replacing fossil-derived polyols in PU networks. As mentioned earlier, xylose is the primary monomeric carbohydrate identified in the THF-soluble fraction after hydrolysis, accounting for 23 ± 3% of the biomass. Considering that pure oregonin contains 31% xylose, we can infer that the 23% xylose content corresponds to the 74% oregonin content in the extractives, which aligns with the UHPLC-ELSD results. Further analysis revealed the presence of low and high molecular weight proanthocyanidins (PAC), constituting 23% of the THF-soluble fraction of the extractives (referred to as “black alder bark-sourced bio-polyol”).

**Figure 2:**
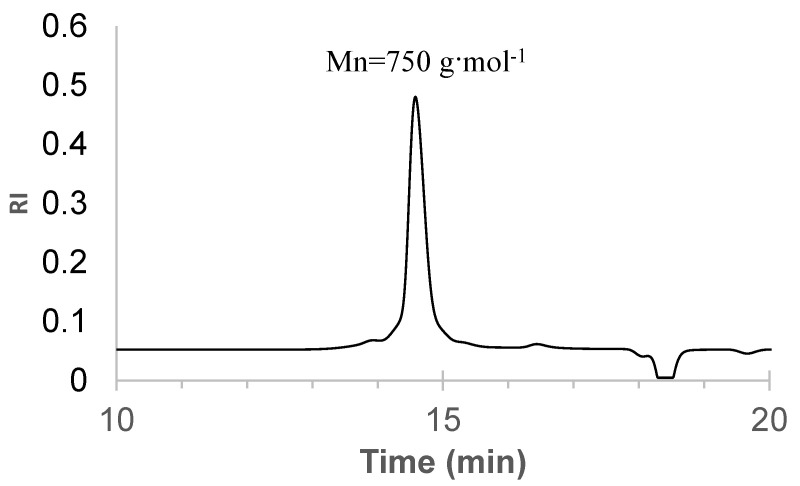
Gel Permeation Chromatography (GPC) of black alder bark-sourced bio-polyol.

The presence of relatively high molecular weight proanthocyanidins (PAC) and carbohydrates in the composition of the bark-sourced macromonomer is responsible for the appearance of high molecular weight peaks on the GPC chromatogram, resulting in a higher average number molecular weight (Mn = 750 g∙mol^−1^) for the bark-sourced bio-polyol compared to the dominant component, oregonin (Mn = 478 g·mol^−1^) (Figure 2).

The dominant peak, corresponding to Mn = 750 g·mol^−1^, occupies 96% of the chromatogram. These GPC results indicate that low molecular weight/oligomeric compounds predominate in the composition of the extractives.

The functionality of the bio-polyol was investigated using independent methods such as NMR ^31^P and wet chemistry (Table 3).

Similar OH group content was determined using both methods. In PU chemistry, the OH number of polyols is typically measured by esterifying them with organic acid and subsequently conducting alkali titration [36]. Therefore, the results from the wet chemistry analysis were utilized to develop the composition of the polyurethane (PU).

One of the main advantages of ^31^P NMR is its ability to provide comprehensive information regarding the origin of phenolic OH groups, which serves as an additional tool for clarifying the composition of biomass [33]. The ^31^P NMR results indicate that phenolic groups in polyol are located mainly in catechol subunits of oregonin. Theoretically, the portion of aliphatic groups in oregonin is 0.43, which is some lower than was detected in polyol (Table 3). This can be explained by the presence of an admixture of carbohydrates of non-oregonin origination enriched with aliphatic OH. The presence of polyol in an almost equal portion of aliphatic OH and phenolic groups differing by reactivity with isocyanate allow us to propose that controlling its conversion may be achieved by using different catalysts [6,37,38]. And at the given catalyst used, the different extent of phenolic and aliphatic OH conversion will be achieved, influencing, therefore, the properties of PU.

### 3.2. Formulation and Structural Characteristics of Polyurethane Networks Depending on the Content of Bark-Sourced Bio-Polyol

#### 3.2.1. Crosslink Density of Polyurethane Networks versus Composition

The equivalent weight (EW) of the polyol is defined as the weight in grams that contains one mole of OH groups [36]. The EW of bio-polyol is more than three times lower than that of the commercial polyol PEG 400: 66.2 g·mol^−1^ and 200 g·mol^−1^, respectively. Therefore, incorporating 30% bio-polyol in the PU composition allows for the substitution of over 90% of PEG 400 at an equimolar NCO/OH ratio (Table 4). Additionally, reducing the EW of the bio-polyol/PEG 400 mixture compared to PEG 400 increases the proportion of isocyanate in the PU composition. When PEG 400 was completely replaced by bio-polyol while maintaining an NCO/OH ratio of 1.0, the resulting PU composition had a bio-polyol content of 33.2%. To increase the proportion of bio-polyol in the PU up to 50%, the NCO/OH ratio was decreased to 0.5 (sample No. 7, Table 4). This adjustment also helped prevent material fragility at peeling from the mold. Formulations containing 33.2% bio-polyol and 66.8% isocyanate contents demonstrated significant destruction when the samples were peeled from the PTFE mold. This can be attributed to the absence of soft segments and the high content of isocyanate.

Crosslink density (XLD), defined as the number of crosslinks per unit volume or weight of the polymer, is one of the fundamental characteristics of thermosetting polymers that directly influence their mechanical and thermal properties [39,40,41,42,43,44]. In this study, the effect of a high-functional bio-polyol on the crosslink density of the PU network was calculated based on the equivalent weight (EW) and number-average molecular weight (Mn) of each PU component, assuming complete NCO conversion using Equations (1)–(3) as described in [40].

(1) $$XLD=\frac{\sum_{i=1}^{n}X_{i}}{2{EW^{\prime }}_{i}}$$

where *XLD* is the crosslink density of PU the network, mol·g^−1^.

*X_i_*—the weight portion of each component in the PU composition, dimensionless.

*EW′_i_*—the adjusted equivalent weight for each component, accounting for the formation of infinite molecular weight, g mol^−1^.

(2) $$\frac{1}{{EW^{\prime }}_{i}}=\frac{1}{{EW}_{i}}-\frac{2}{{Mn}_{i}}$$

where *EW_i_*—the equivalent weight of each component, g mol^−1^.

*Mn_i_*—number-average molecular weight of each component, g·mol^−1^.

If the polyol was not completely reacted due to a non-stoichiometric ratio (NCO/OH < 1), the adjustment for the polyol was made using the following equation:

(3) 1EWi′=NCOOHEWi−2Mni

In addition, the crosslink density (*XLD*) was recalculated as the molecular weight per mole of elastically networked chains in Da (*Mc*), using Equation (4).

(4) $$M_{c}=\frac{2}{XLD}$$

The performed calculations have shown a permanent increase in the crosslink density of the PU as the bio-polyol content in the composition increases while maintaining a fixed NCO/OH ratio (see Table 4). Introducing 30% bio-polyol in the PU composition resulted in a more than six-fold increase in crosslink density compared to the initial PU composition containing 60% PEG 400. The crosslink density of the PU with 50% bio-polyol content, despite having a non-stoichiometric NCO/OH ratio, was similar to that of the PU with 20% bio-polyol content at an equimolar NCO/OH ratio.

To experimentally assess the crosslinking effect of the bio-polyol, the initial PU samples and the PU samples containing 30% bio-polyol (which exhibited the highest crosslink density according to the earlier calculations) were subjected to a one-week leaching process in a THF solution, where each component of the PU is easily soluble. The soluble portion, known as the non-crosslinked sol fraction, was determined following the methodology described in the Experimental section. The sol fraction content for the bio-polyol-free PU sample and the sample containing 30% bio-polyol was found to be 6.0 ± 0.5% and 1.2 ± 0.2%, respectively, indicating a crosslinking effect of the bio-polyol. The data obtained for PU networks synthesized with DBTDL and DABCO catalysts showed similar trends, considering the standard deviation from repeated measurements (Figure S2).

#### 3.2.2. FTIR Spectra of PU Networks

**Figure 3:**
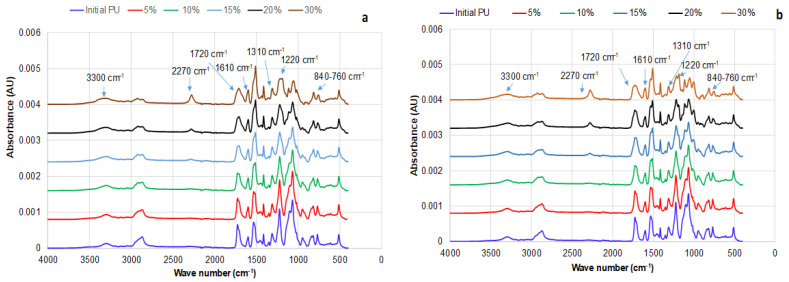
FTIR Spectra of PU with varied bio-polyol content in the composition synthesized with DBTDL (**a**) and DABCO (**b**) catalyst.

The FTIR spectra of the obtained PU samples exhibit characteristic absorbance regions corresponding to urethane bonds. Specifically, the spectra show absorbance peaks at the following wavenumbers: 3200–3300 cm^−1^, attributed to the N-H stretch in urethane groups; 1720 cm^−1^, indicating carbonyl stretch in urethane bonds; 1611 cm^−1^, representing C-N stretch; 1310 cm^−1^, associated with coupled bending of N-C-O and CH_2_; 1220 cm^−1^, reflecting asymmetric stretching of N-CO-O; and 845–760 cm^−1^, attributed to various C-N vibrations (Figure 3a,b) [45,46,47].

The FTIR spectra of PU synthesized with both DBTDL and DABCO catalysts exhibit remarkable similarity. In both sets of spectra, there is no absorbance observed at around 2270 cm^−1^, which is characteristic of free NCO. This absence of the peak is observed for bio-polyol contents up to 15%. However, at higher concentrations (≥15%), the presence of this peak indicates incomplete NCO conversion, suggesting that the crosslinking may be slightly lower compared to the theoretical calculation. However, the intensity of these peaks is very low, indicating that the majority of OH groups reacted with isocyanate in the presence of both catalysts, which was also supported by leaching tests (Figure S2).

**Figure 4:**
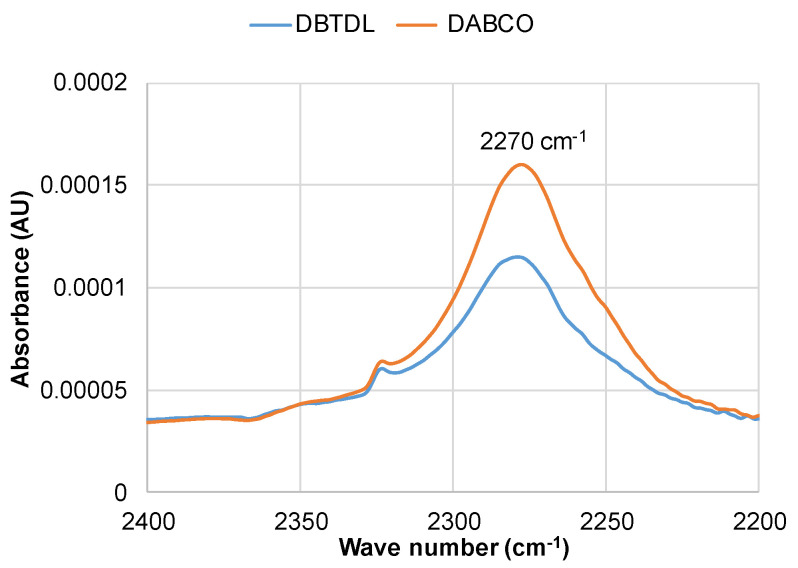
FTIR spectra region displaying absorbance at 2270 cm^−1^ for PU networks with 20% bio-polyol content, cured with DBTDL and DABCO catalysts.

In the PU composition with a specific bio-polyol content, PU synthesized with the tin organic catalyst shows a higher concentration of urethane bonds. These findings are supported by the higher absorbance intensity observed at 2270 cm^−1^, indicating non-reacted NCO groups in PU synthesized with DABCO compared to that synthesized with the DBTDL catalyst (Figure 4).

This can be attributed to the greater catalytic activity of tertiary amines, which act as basic catalysts, in promoting the interaction between acidic phenolic groups and the isocyanate, as well as the remaining unreacted aliphatic hydroxyl groups. Conversely, in the case of DBTL, which exhibits higher catalytic activity towards aliphatic groups, a more favorable balance is achieved between the conversion of both aliphatic hydroxyl and phenolic groups, resulting in the presence of some non-reacted phenolic groups.

### 3.3. Thermal Properties of PU Networks Depending on the Content of Bark-Sourced Bio-Polyol

#### 3.3.1. Glass Transition Temperature

The glass transition temperature (Tg) reflects the segmental mobility of the main polymeric chains and is a phenomenon observed in amorphous polymers, including PU elastomers [10,48,49]. At the glass transition temperature, the heat energy absorbed by the polymer chains begins to exceed the energy of intermolecular bonds. As a result, at this temperature, the mobility of the polymer chains increases, causing the structure to transition from a glassy state with frozen chain mobility to a highly elastic state with enhanced mobility of polymeric chains. This transition leads to a dramatic change in the mechanical characteristics of the polymer, including a decrease in rigidity and mechanical strength and an increase in flexibility accompanied by an increase in heat capacity. This physical and chemical parameter correlates with the heat resistance of the polymer, which is an important technological characteristic reflecting the polymer’s ability to retain its deformation characteristics at elevated temperatures.

**Figure 5:**
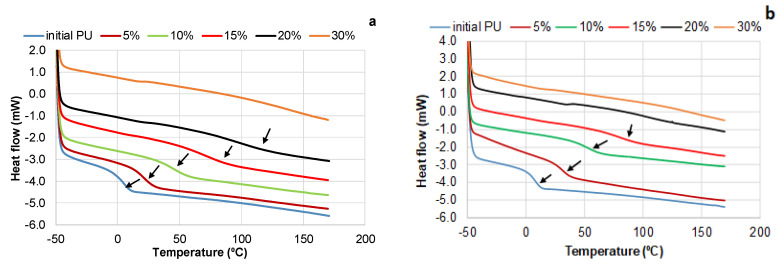
DSC curves at glass transition of PU networks synthesized in the presence of DBTDL (**a**) and DABCO (**b**) catalysts. (The position of Tg is marked by arrows).

The results of this study have shown that together with increasing the bio-polyol content up to 20% for PUs synthesized with DBTDL catalyst and 15% for PUs synthesized with DABCO catalyst, the Tg of PU elastomers is increasing while the heat capacity jump (ΔCp) is decreasing (Table 5). This can be explained by a decrease in the free polymer volume and free molecular motion in the PU structure, attributed to an increase in both crosslink density and the content of bulk aromatic units in PU networks [10,11,50,51]. In all cases, only a single glass transition range was observed, which is typical for amorphous phases that achieve miscibility through specific (hydrogen bonding) or non-specific interchain interactions (Figure 5) [52,53]. Consequently, phase separation does not occur.

In the range of bio-polyol content of 5–15%, the Tg of PUs synthesized in the presence of amine catalysts was higher, but the heat capacity jump was lower compared to PUs synthesized with tin organics. The crosslink density (see Section 3.2.1.) and the content of soft (PEG 400) and hard (PMDI plus bio-polyol) segments are the same for both series of PUs at a given bio-polyol content. Therefore, it can be assumed that in the presence of DABCO, the higher energy of intermolecular interaction, including covalent and physical bonds, was achieved in the PU networks. No glass transition was observed in the PU networks synthesized with the DBTDL catalyst when the bio-polyol content was 30% at an equimolar NCO/OH ratio. Similarly, for the PUs synthesized with the DABCO catalyst, no glass transition was detected at a bio-polyol content exceeding 15%. In these cases, the energy of interaction within the PU network was significantly high, and the free polymer volume was extremely low, preventing the amorphous rigid structure of the PU from transitioning to a more flexible structure when heated up to 180 °C. Further heating initiated thermal degradation processes, making it impossible to detect the glass transition of PU networks at high contents of bio-polyols.

It is well known that the NCO/OH ratio is an effective tool for controlling the crosslinking density of PU networks [54]. By reducing the NCO/OH ratio to 0.5, it was possible to increase the bio-polyol content in PU networks up to 50% (Table 5). In this case, the calculated molecular weight (Mc) was 1180 Da, which was close to that of a PU network containing 20% bio-polyol at a stoichiometric NCO/OH ratio (Table 5). As a result, similar values of Tg (105–110 °C) were determined for DBTDL-catalyzed PU networks containing 20% and 50% bio-polyol. These findings indicate that the introduction of bio-polyol is an effective method for increasing the heat resistance of PU elastomers. In the case of DABCO catalyst compared to DBTDL, similar segmental mobility in PU networks can be achieved by using a composition with reduced content of bio-polyol.

#### 3.3.2. Thermal Degradation of PU Networks in Air

**Figure 6:**
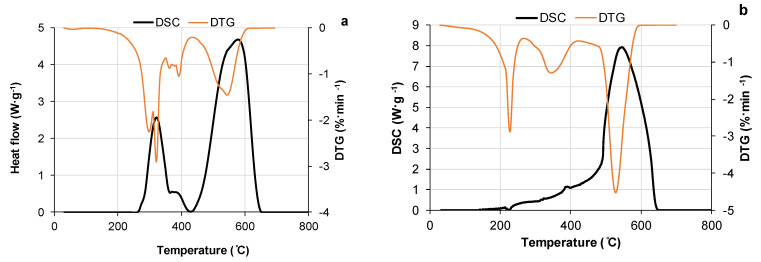
DSC and DTG curves of PU networks in the air: (**a**) initial and (**b**) with 30% of bio-polyol content (catalyst DABCO).

The non-isothermal TG/DTG/DSC analysis of PU networks indicates the presence of two major stages in their thermal degradation process in an oxidative environment (Figure 6). According to literature data, the first stage at lower temperatures represents the complex multistep destruction of the molecular structure of PU, accompanied by the formation of volatiles such as tar and gas [55]. These volatiles undergo oxidation in the presence of air, releasing heat and leading to the formation of solid residues through condensation reactions. In the case of the PUs under study, this stage occurs at approximately 200–450 °C (Figure 6). The second stage at higher temperatures is attributed to the heterogeneous oxidation and combustion of the char residue formed from the molecular destruction of PU [55,56]. For the materials under study, the oxidation of char begins at around 425 °C and completes at approximately 650 °C (Figure 6).

The low-rate weight loss during the thermal degradation of the initial PU starts at temperatures of approximately 250 °C for materials synthesized with both DBTDL and DABCO catalysts (Table 6). The introduction of 15–30% bio-polyol at the same NCO/OH ratio shifts the temperature of the start of degradation to a lower range, up to 189 °C. The same tendency is observed for the temperature of maximum degradation rate (Table 6). This can be explained by the lower thermal stability of urethane bonds formed by phenolic groups and aryl isocyanate, which degrade at around 120 °C, in comparison with urethane bonds formed by the reaction of aryl isocyanate with aliphatic OH, which degrades at around 200 °C [54,55,57,58]. At the same time, the introduction of bark-sourced polyol in the PU network decreases the rate of degradation of the material, with a higher effect for a higher bio-polyol content (Table 6).

For a deeper understanding of the thermal degradation behavior of the PUs under study, thermal analysis was conducted on the components used as building blocks for the PUs. Among the three components of the PU networks, bio-polyol exhibited the lowest temperature for degradation initiation at 168 °C, compared to 215 °C for PEG 400 and 232 °C for PMDI, and it also showed the highest rate of degradation at lower temperatures (Table 7 and Figure S3). This explains the observed shifts to lower temperature regions in the first stage of thermal degradation for PUs incorporating more than 10% of bark-sourced polyols (Table 6). At the same time, the average rate of bio-polyol degradation in the zone of volatile formation was 2.6 and 4.0 times lower compared to that of PEG 400 and PMDI, respectively (Table 7).

As a result of bark-sourced polyol thermal degradation behavior, the average rate of PU degradation decreased with increasing content of bio-polyol, resulting in a decrease in mass loss during the volatile formation stage. Simultaneously, the weight loss in the high-temperature zone responsible for char combustion increased (Figure 6). These facts can be considered positive from the perspective of reducing the flammability of PUs, as flammability is associated with the formation of flammable volatiles during initial decomposition [55]. On the contrary, char formation reduces the amount of volatile fuel, which contributes to the flammability of the polymer. Additionally, the char provides a thermally insulating layer that prevents heat and fire transfer inside the material [59].

For a more detailed study of the effect of bio-polyol on PU degradation in terms of weight loss and heat released during the volatile formation and char combustion stages, two approaches were used to calculate the processing conversion. In the first approach, TG data were utilized, and the weight conversion *(X_w_)* was calculated by Equation (5) [56].

(5) $$X_{w}=\frac{W_{0}-W_{t}}{W_{0}-W_{f}}$$

where:

*X_w_*—conversion by weight, dimensionless

*W_*0*_*—the initial weight of the sample, mg

*W_t_*—sample weight at any moment of time, mg

*W_f_*—final weight of the sample, mg

The final weight of samples consisted of ≤1.5% of their starting weight because of ash content in them.

The second approach was based on DSC data, and the conversion by heat (*X*_Δ*H*_) was calculated using the basic equation of differential calorimetry (6) [60]:

(6) $$X_{∆H}=\frac{\int_{0}^{t}q\left(t\right)dt}{∆H}$$

where:

*X*_Δ*H*_—conversion by heat, dimensionless

*q*(*t*)*—*heat flow at any moment of time, W·g^−1^

∆*H*—total heat released, kJ·g^−1^

The specific heat released as a result of thermo oxidative degradation of the PU network and its constituents was 13 ± 2 kJ·g^−1^.

**Figure 7:**
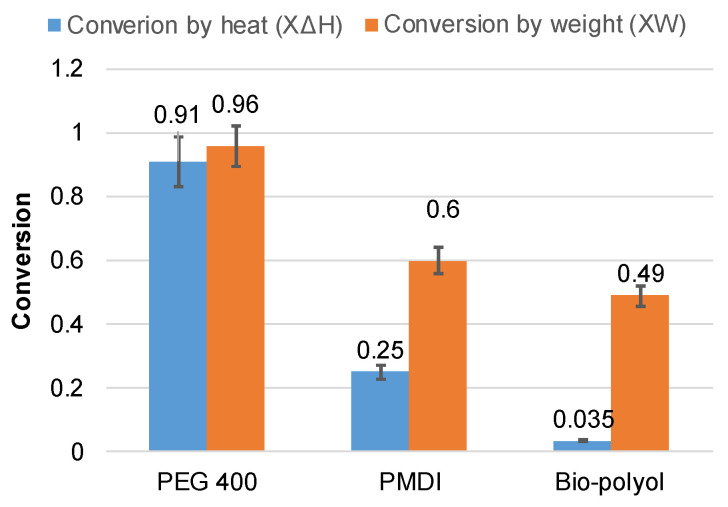
The values of conversions by heat (*X*_∆*H*_) and by weight (*X_w_*) for the volatile formation stage of PU network constituents: PEG 400, PMDI, and bio-polyol.

The calculations conducted for the building blocks have shown that for PMDI and bio-polyol, the conversion by heat during the volatile stage of degradation was significantly lower than the conversion by weight, indicating that the major portion of heat was released as a result of char oxidation. However, for PEG 400, very similar values were obtained for both types of conversion. In this case, at the end of the volatile formation stage, the achieved values of conversion by weight and by heat were 0.96 and 0.91, respectively (Figure 7 and Figure S3).

**Figure 8:**
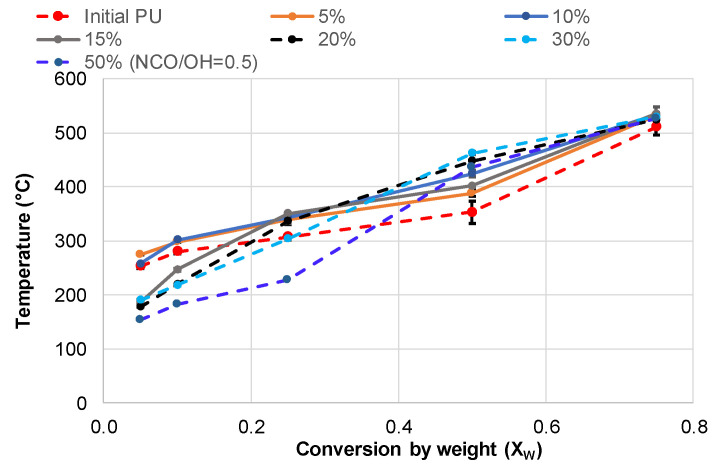
Conversion by weight (*X_w_*) vs. degradation temperature for PU networks synthesized with DBTDL as a catalyst and varying bio-polyol content.

Among all the components, bio-polyol exhibited the lowest values for both *X*_∆*H*_ and *X_w_*. Only 3.5% of the total heat was released as a result of volatile oxidation in the temperature range of 168–440 °C. Correspondingly, approximately 50% of the bio-polyol was degraded in the high-temperature zone of char oxidation, accounting for 96% of the total heat release. The increase in bio-polyol content and a simultaneous decrease in PEG 400 content led to a decrease in heat released during the volatile formation (Figure 8).

**Figure 9:**
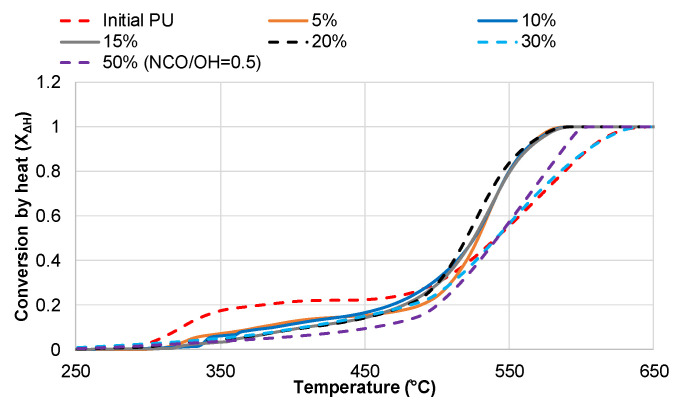
The effect of temperature on the conversion by heat (*X*_∆*H*_) of PU networks synthesized with DBTDL as a catalyst and varying bio-polyol content.

The effect of bio-polyol on the temperature dependence of PU conversion by weight is more complicated. At 5% and 10% bio-polyol content, the PUs exhibit higher thermostability throughout the entire conversion range. However, with further increases in bio-polyol content, the temperature at which degradation begins (*X_w_* = 0.05–0.25) decreases. Nevertheless, for all PU networks containing bio-polyol, *X_w_* ≥ 0.4 is achieved at a higher temperature compared to that observed for the initial PU (Figure 9).

As a result, the conversion by weight of bio-polyol-containing PU in the temperature range of volatile formation was lower. This is obviously the main reason for the decrease in heat output during the volatile oxidation step of bio-polyol-containing PU networks.

**Figure 10:**
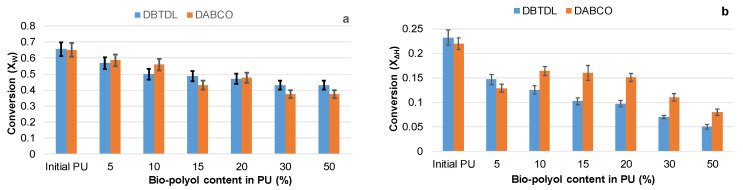
The achieved conversion values by weight (**a**) and by heat (**b**) at the end of the volatile formation stage for PU networks, depending on the bio-polyol content and the type of catalyst used.

The values of *X_w_* and *X*_∆*H*_ achieved at the end of the volatile formation stage of PU degradation were calculated for compositions synthesized with both catalysts (Figure 10).

As shown, a similar decrease in volatile release was observed with increasing bio-polyol content for PU networks synthesized with both catalysts. However, the decrease in heat released during volatile oxidation was more pronounced for PU synthesized with DBTDL. This can be explained by the complete interaction between the acidic phenolic groups of bio-polyol and isocyanate in the presence of amine catalysts, which act as bases in the formation of an active complex. Consequently, the radical scavenging activity of phenolic compounds in bio-polyol is diminished. In contrast, with DBTDL acting as a Lewis acid and catalyzing the reaction between the less acidic aliphatic OH and NCO, a portion of free polyphenolic compounds may remain. This allows for radical scavenging activity, thereby reducing oxidation processes and the amount of heat released.

In conclusion, it can be stated that the introduction of bio-polyols into PU networks acts as a crosslinker and promotes char formation. As a result, the amount of combustible volatiles decreases, reducing the heat liberated during the initial stages of degradation. The process of PU thermal degradation is shifted towards char oxidation, which occurs at higher temperatures. The obtained results suggest that the introduction of bio-polyols can decrease the flammability of PU networks. Further investigations using cone calorimeter tests are currently being planned.

### 3.4. The Tensile Properties of PU Networks Depending on Bio-Polyol Content

As mentioned above, the introduction of bio-polyol into the PU composition leads to a decrease in the soft segment content in the PU network, an increase in the content of bulk aromatic units, and an increase in crosslink density. These structural parameters play a crucial role in the mechanical characteristics of PU networks [9,12,13]. Consequently, significant changes in the deformation characteristics of PU networks containing bio-polyol were observed in comparison to the initial PU.

**Figure 11:**
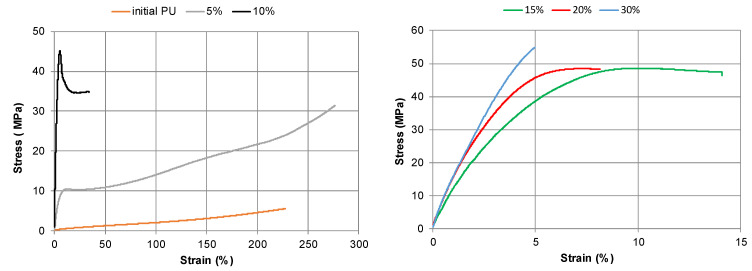
Stress-strain curves of PU networks synthesized with DBTDL catalyst depending on bio-polyol content.

As stated in the experimental section, the temperature during the tensile testing of the PU samples was 22 ± 1 °C. Consequently, the glass transition temperature of the initial PU synthesized with both DBTDL and DABCO catalysts was below the testing temperature (Table 5). This resulted in stress-strain curves of the initial PU typical for rubbery polymers, where low stress was observed at high deformations (Figure 11).

**Figure 12:**
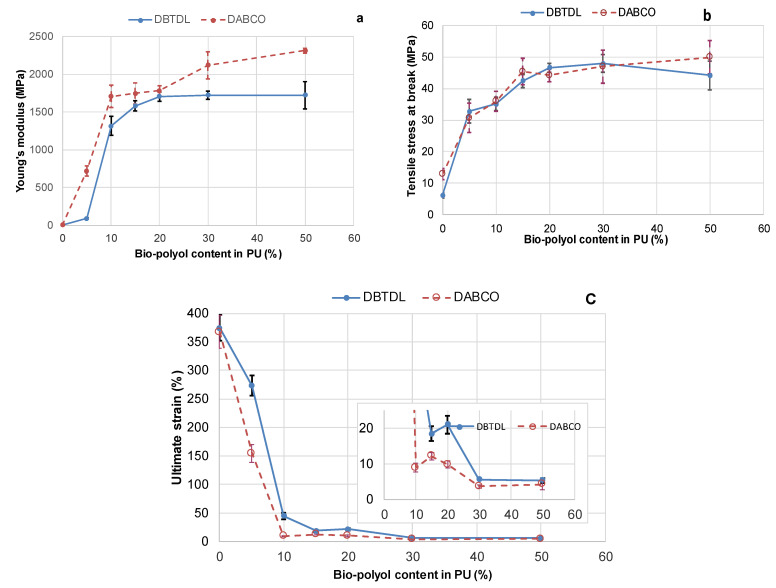
Dependence of tensile characteristics, including Young’s modulus (**a**), strength at break (**b**), and ultimate strain (**c**), of PU networks synthesized with DBTDL and DABCO catalysts on bio-polyol content.

The other PU samples containing bio-polyol were in a glassy state during testing, and their behavior during tensile testing differed significantly from that of the initial PU. An increase in bio-polyol content up to 5–10% resulted in the appearance of a forced rubbery elasticity region on the deformation curves, which is typical for crosslinked elastomers in a glassy state. Drastic increases in Young’s modulus and stress at break, along with simultaneous significant decreases in ultimate strain, were observed (Figure 11 and Figure 12).

Further increases in bio-polyol content up to 20% led to a decrease in the high elastic deformation zone, a moderate decrease of ultimate strain, and a gradual increase in material rigidity and strength (Figure 11 and Figure 12). When the bio-polyol content was increased up to 30%, a tenfold decrease in soft segment content compared to the initial PU network was observed. For this composition, the highest crosslinking of the PU network was calculated (Table 3). In this case, the high elasticity deformation disappeared, and fragile destruction was observed throughout the entire range of deformation, accompanied by a decrease in ultimate strain while maintaining constant strength and modulus values. Some increases in modulus values were observed with increasing bio-polyol content of up to 30% for PU networks synthesized with DABCO catalysts (Figure 12a).

A comparison of the tensile characteristics of PU networks synthesized with different catalysts reveals higher values of Young’s modulus for PU synthesized with DABCO catalyst compared to those synthesized with DBTDL across all ranges of bio-polyol concentration. Additionally, there is a slight decrease in ultimate strain while maintaining similar values of strain at break (Figure 12).

The higher crosslink density observed in PU networks synthesized with DABCO catalysts, compared to those synthesized with DBTDL, can likely explain the higher values of Young’s modulus in the networks synthesized with DABCO. This is attributed to the increased involvement of phenolic groups in the formation of urethane bonds in the presence of a DABCO catalyst. The results demonstrate that increasing the bio-polyol content up to 50% while simultaneously decreasing the NCO/OH ratio to 0.5 does not significantly influence the tensile properties of PU networks containing 30% bio-polyol and an equivalent concentration of PMDI. Both catalysts resulted in rigid but fragile PU samples at 30% and 50% bio-polyol content. However, at 15–20% bio-polyol content, PU networks with high strength and sufficient flexibility (ultimate strain of 10–20%) were obtained.

Based on these findings, it can be hypothesized that increasing the bio-polyol content in PU within the range of 30% to 40% and simultaneously decreasing the NCO/OH ratio may potentially reduce the fragility of the resulting PU while maintaining a higher bio-polyol content. These experiments will be conducted as part of further investigations.

## 4. Conclusions

A new bio-polyol, composed of 74% diarylheptanoid oregonin, 26% oligomeric flavonoids, and carbohydrates, with an average molecular weight (Mn) of 750 g·mol^−1^ and an OH content of 15 mmol·g^−1^ evenly distributed between aliphatic and phenolic groups, was derived from black alder bark using green chemistry principles. It was successfully incorporated into PU networks through copolymerization with isocyanate, replacing up to 100% of the fossil-based polyol PEG 400. Depending on the NCO/OH ratio, the bio-polyol content in the PU composition reached a maximum of 50%. Complete conversion of NCO groups was achieved with approximately 15% bio-polyol content, while the majority of NCO conversion occurred at higher bio-polyol contents. Calculations and experimental data demonstrated the crosslinking effect of the bark-sourced polyol, effectively enhancing the heat resistance of PU elastomers. The properties of the material were regulated by catalysts used for PU network curing, which controlled the reactivity of various hydroxyl groups. Notably, the PU network cured with the amine catalyst DABCO exhibited the most significant increase in Tg. This can be attributed to higher interchain interaction energy involving covalent bonds of fully reacted, more acidic phenolic groups and non-covalent bonds of some unreacted aliphatic hydroxyl groups, such as hydrogen bonds. The bark-sourced building blocks promoted char formation under high temperatures and oxidative stress, reducing combustible volatiles and limiting heat release during macromolecular network degradation. This shift towards char oxidation at higher temperatures suggests a potential decrease in flammability during actual combustion tests of bio-polyol-based PUs. The PUs displayed significantly higher strength and rigidity while maintaining sufficient flexibility when the bark-sourced polyol content was not more than 20%. However, exceeding this threshold led to the formation of rigid and brittle materials. Considering the high functionality of the bark-derived bio-polyol, further optimization of PU network characteristics will involve simultaneously increasing the bio-polyol content and decreasing the NCO/OH ratio, achieving a suitable balance between material strength and rigidity on the one hand and sufficient elongation at the break on the other.

## Figures and Tables

**Table 1:** UPLC-ELSD gradient system.

T, min	A, %	B, %
0	95	5
0.5	95	5
17	5	95
18	5	95
18.5	95	5
20	95	5

**Table 2:** Monomeric carbohydrate content (%) in raw extract and its fractions after complete hydrolysis, as analyzed by GC.

Sample	Glc ^1^	Gal ^2^	Man ^3^	Xyl ^4^	Ara ^5^	Rha ^6^	Sum
Raw extract	15.8 ± 0.7	1.5 ± 0.1	3.7 ± 0.2	15.1 ± 0.6	2.0 ± 0.4	0.12 ± 0.3	38.1± 2.3
THF-soluble fraction	10.5 ± 0.6	n.d	1.3 ± 0.2	23.0 ± 0.7	0.32 ± 0.10	n.d	35.1± 1.6
THF-insoluble fraction	23.7 ± 1.6	3.0 ± 0.3	7.5 ± 0.6	6.5 ± 0.3	3.9 ± 0.1	0.3 ± 0.1	44.9 ± 3.0

^1^ glucose; ^2^ galactose; ^3^ mannose; ^4^ xylose; ^5^ arabinose; ^6^ rhamnose.

**Table 3:** Content of various OH groups in black alder bark-sourced bio-polyol as determined by wet chemistry and ^31^P NMR methods.

Functional Group	Content (mmol·g^−1^) Wet Chemistry ^31^P NMR	
Aliphatic OH	6.8 ± 0.7	8.4
C5 substituted OH	-	0.1
Catechol OH	-	8.2
P-hydroxyphenyl OH	-	0.4
Total phenolic	7.7 ± 0.5	8.7
Carboxylic OH	0.58 ± 0.05	0.2
Total OH groups (∑OH)	15.1 ± 1.3	17.2
OH aliph/∑OH	0.45	0.49

**Table 4:** Content of various OH groups in black alder bark-sourced bio-polyol as determined by wet chemistry and ^31^P NMR methods.

Sample	Content in PU (%) Bio-Polyol PEG 400 PMDI			Substitution of PEG 400 (%)	Crosslink Density XLD·10^−4^ (mol·g^−1^) Mc (Da)	
1	0	60	40	0	0.38	5230
2	5	51	44	15.0	0.73	2740
3	10	42	48	30.0	1.08	1850
4	15	33	52	45.0	1.43	1400
5	20	24	56	60.0	1.77	1120
6	30	6	64	90.0	2.47	810
7	50	0	50	100	1.69	1180

NCO/OH = 1.0 (No. 1–6); NCO/OH = 0.5 (No. 7).

**Table 5:** Tg values and corresponding heat capacity jump (ΔCp) for PUs synthesized with different catalysts depending on the bio-polyol content.

Composition	Bio-Polyol Content in PU (%)	DBTDL Tg (°C) ΔCp (J·g^−1^·K^−1^)		DABCO Tg (°C) ΔCp (J·g^−1^·K^−1^)	
1	Initial PU	4.9 ± 0.5	0.50 ± 0.03	5.6 ± 0.5	0.60 ± 0.05
2	5	21.5 ± 1.5	0.58 ± 0.06	32.0 ± 2.0	0.45 ± 0.02
3	10	45.7 ± 0.8	0.42 ± 0.03	53.5 ± 3.0	0.30 ± 0.03
4	15	74.6 ± 2.2	0.32 ± 0.02	82.9 ± 2.3	0.25 ± 0.02
5	20	103.9 ± 6.2	0.16 ± 0.01	n.d	n.d
6	30	n.d	n.d	n.d	n.d
7	50	110.7 ± 4.8	0.20 ± 0.03	n.d	n.d

NCO/OH = 1.0 (samples No. 1–6); NCO/OH = 0.5 (sample No. 7).

**Table 6:** Parameters of the volatile formation stage during thermal oxidative degradation of PU networks synthesized using different catalysts, depending on the content of bio-polyol in them.

		DBTDL Catalysts			DABCO Catalyst		
Sample	Bio-Polyol Content in PU (%)	Temperature Range (°C)	Tmax ^1^ (°C)	Average Rate (%·min^−1^)	Temperature Range (°C)	Tmax ^1^ (°C)	Average Rate (%·min^−1^)
1	Initial PU	254–436	328 ± 8	1.8 ± 0.1	256–460	396 ± 7	1.8 ± 0.1
2	5.0	275–439	325 ± 4	1.6 ± 0.2	271–458	377 ± 8	1.40 ± 0.2
3	10.0	258–451	331 ± 5	1.2 ± 0.1	234–451	343± 4	1.2 ± 0.1
4	15.0	187–435	387 ± 2	1.0 ± 0.1	180–459	395 ± 5	0.84 ± 0.05
5	20.0	178–442	383 ± 5	0.90 ± 0.07	156–460	402 ± 6	0.87 ± 0.06
6	30.0	189–426	229 ± 5	0.79 ± 0.05	171–424	229 ± 5	0.76 ± 0.10
7	50.0	154–430	207 ± 2	0.74 ± 0.10	156–436	208 ± 3	0.82 ± 0.07

NCO/OH = 1.0 (samples No. 1–6); NCO/OH = 0.5 (sample No. 7); The temperature at which 5% weight loss of the PU samples was achieved was defined as the starting temperature of degradation; ^1^ The temperature at which the maximal rate of volatile formation was achieved.

**Table 7:** Parameters of the volatile formation stage during thermal oxidative degradation of PU network constituents: PEG 400, PMDI, and bio-polyol.

Parameter	PEG 400	PMDI	Bio-Polyols
Temperature range (°C)	215–360	232–520	168–440
Tmax ^1^ (°C)	290 ± 5	268 ± 7	224 ± 8
Average rate (%·min^−1^)	3.2 ± 0.2	2.1 ± 0.1	0.80 ± 0.07

^1^—the temperature at which the maximal rate of volatile formation was achieved.

## Data Availability

The data presented in this study are available on request from corresponding authors.

## Supplementary Materials

The following supporting information can be downloaded at: https://www.mdpi.com/article/10.3390/polym15173503/s1, Figure S1: UHPLC-TOF chromatogram of the black alder bark-sourced bio-polyol; Figure S2: Yield of sol fractions for initial PU networks and those containing 30% of bio-polyol after one week leaching in THF; Figure S3: DTG /DSC curves (a,b,c) and conversion curves (d,e,f) of thermal oxidative degradation of bio-polyol (a,d), PMDI (b,e) and PEG 400 (c,f); Table S1: Identification of compounds presented in bark-sourced bio-polyol.
