# Supplementary material for: Impact of Bark-Sourced Building Blocks as Substitutes for Fossil-Derived Polyols on the Structural, Thermal, and Mechanical Properties of Polyurethane Networks
Source: Polymers (Basel). 2023 Aug 22;15(17):3503. doi: 10.3390/polym15173503 (PMC10490025; doi:10.3390/polym15173503)
Supplement: Supplementary file 1 [file polymers-15-03503-s001.zip › polymers-2532483-supplementary.pdf]

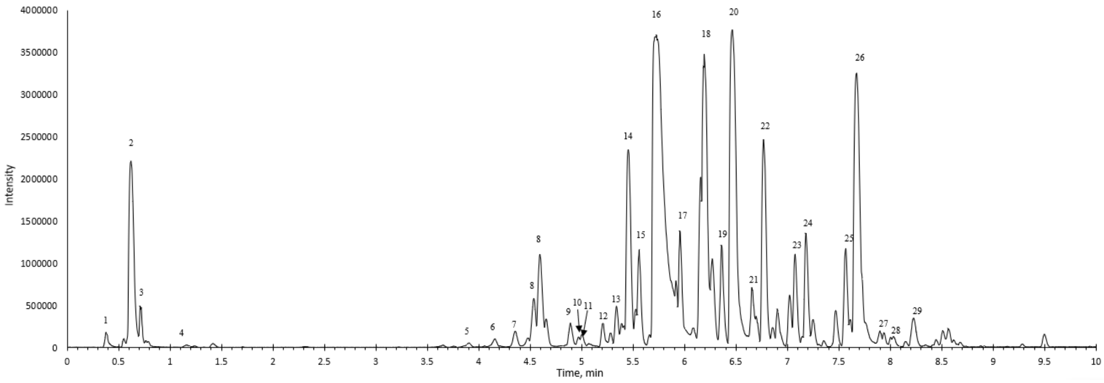

Figure S1 UHPLC-TOF chromatogram of the black alder bark-sourced bio-polyol

Table S1. Identification of compounds presented in bark-sourced bio-polyol

Peak	Retention time, (min)	Compound
1	0.38	Carbohydrates
2	0.62	Myzodendrone
3	0.71	Quinic acid
4	1.16	Quinic acid
5	3.90	(Epi)catechin- (epi)catechin
6	4.16	Catechin
7	4.36	Coumaroylquinic acid
8	4.60	Epi)catechin- (epi)catechin
9	4.89	Epi)catechin- (epi)catechin- (epi)catechin
10	4.98	Coumaroylquinic acid
11	5.00	3-O-galloyl- (epi)gallocatechin- (epi)gallocatechin
12	5.21	Epi)catechin- (epi)catechin- (epi)gallocatechin
13	5.34	catechin-3-O-gallate
14	5.45	Hirsutenone hexoside
15	5.56	Hydroxyoregonin
16	5.73	Oregonin
17	5.96	Oregonin
18	6.19	Rubranol C
19	6.37	Aceroside VII
20	6.46	Rubranoside A
21	6.65	Hirsutenone derivative
22	6.77	Rubranol xyloside
23	7.07	Blank
24	7.18	Blank
25	7.47	1- (4-hydroxyphenyl)-7- (3.4-dihydroxyphenyl) heptan-3-one-5-O-pentoside

26	7.67	Hirsutenone
27	7.90	Hirsutenone
28	8.03	Hirsutenone
29	8.22	Gingerol

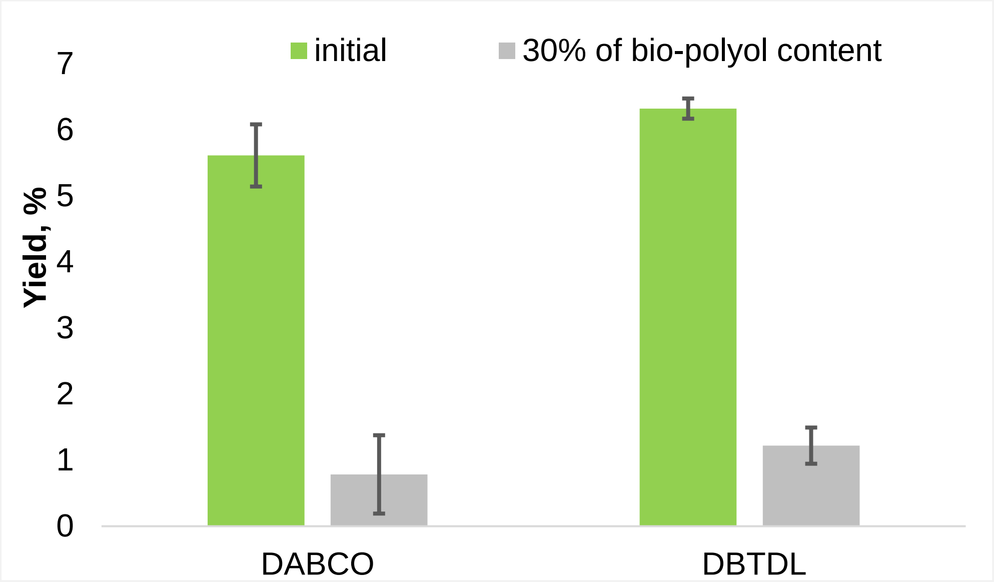

Figure S2. Yield of sol fractions for initial PU networks and those containing 30% of bio-polyol after one week leaching in THF.

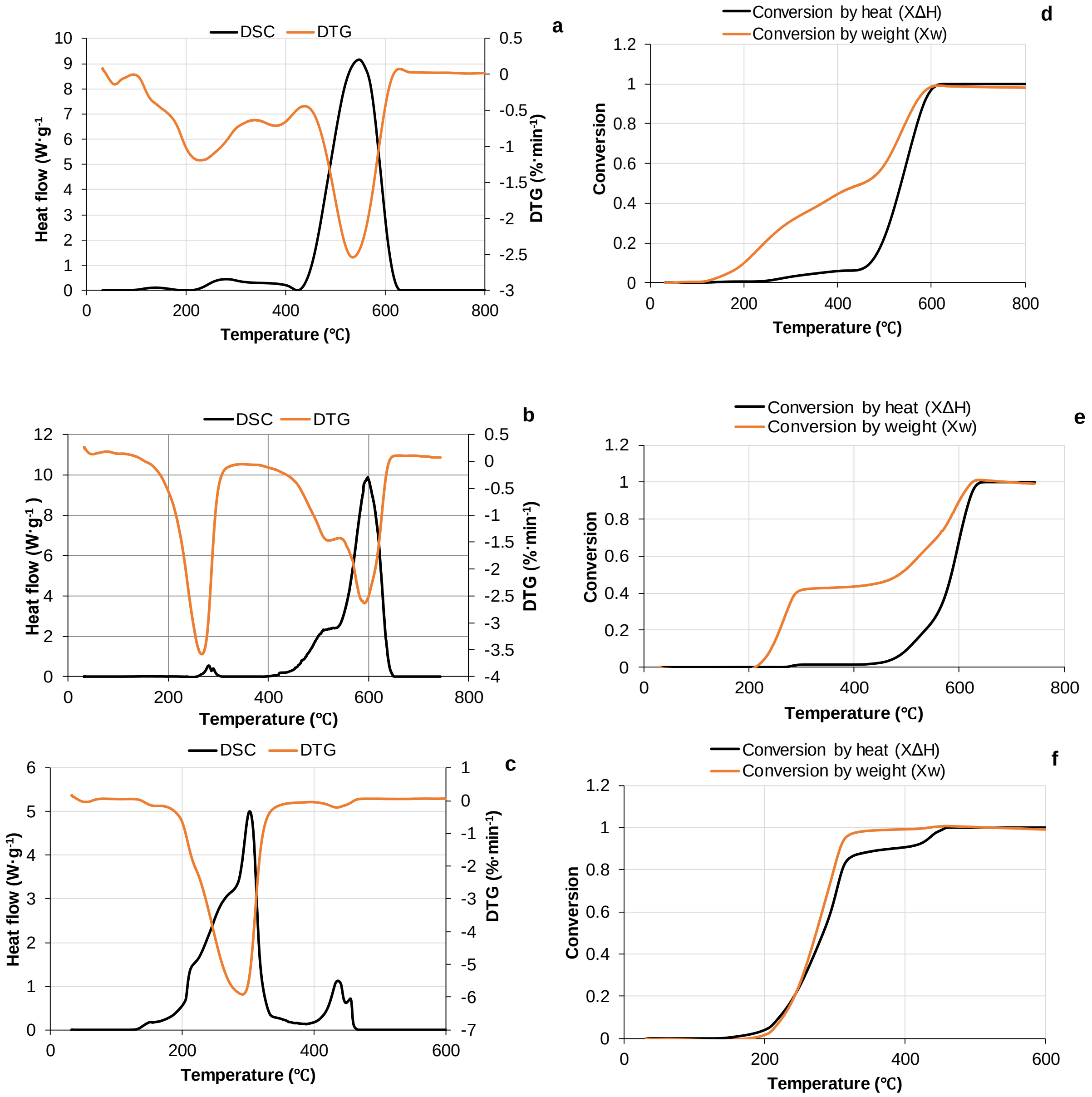

Figure S3. DTG /DSC curves (a, b, c) and conversion curves (d,e,f) of thermal oxidative degradation of bio-polyol (a;d), PMDI (b,e) and PEG 400 (c;f).
